# Load Resistance Optimization of Bi-Stable Electromagnetic Energy Harvester Based on Harmonic Balance

**DOI:** 10.3390/s21041505

**Published:** 2021-02-22

**Authors:** Sungryong Bae, Pilkee Kim

**Affiliations:** 1 Department of Fire Protection and Disaster Management, Chosun University, 309 Pilmun-daero, Dong-gu, Gwangju 61452, Korea; sbae@chosun.ac.kr; 2 School of Mechanical Design Engineering, College of Engineering, Jeonbuk National University, 567 Baekje-daero, Deokjin-gu, Jeonju-si, Jeollabuk-do 54896, Korea; 3 Eco-Friendly Machine Parts Design Research Center, Jeonbuk National University, 567 Baekje-daero, Deokjin-gu, Jeonju-si, Jeollabuk-do 54896, Korea

**Keywords:** electromagnetic energy harvester, bi-stable oscillator, load resistance optimization, frequency response analysis, harmonic balance method

## Abstract

In this study, a semi-analytic approach to optimizing the external load resistance of a bi-stable electromagnetic energy harvester is presented based on the harmonic balance method. The harmonic balance analyses for the primary harmonic (period-1*T*) and two subharmonic (period-3*T* and 5*T*) interwell motions of the energy harvester are performed with the Fourier series solutions of the individual motions determined by spectral analyses. For each motion, an optimization problem for maximizing the output power of the energy harvester is formulated based on the harmonic balance solutions and then solved to estimate the optimal external load resistance. The results of a parametric study show that the optimal load resistance significantly depends on the inductive reactance and internal resistance of a solenoid coil––the higher the oscillation frequency of an interwell motion (or the larger the inductance of the coil) is, the larger the optimal load resistance. In particular, when the frequency of the ambient vibration source is relatively high, the non-linear dynamic characteristics of an interwell motion should be considered in the optimization process of the electromagnetic energy harvester. Compared with conventional resistance-matching techniques, the proposed semi-analytic approach could provide a more accurate estimation of the external load resistance.

## 1. Introduction

Energy harvesting is a series of processes that convert ambient energy (vibration, sunlight, wind, raindrop, earth heat, human motion, etc.), usually unused and wasted, into electric energy that is stored in a capacitor or used to supply power to micro-devices. The energy harvesting technology is considered a promising alternative to chemical batteries for autonomous wireless microdevices. In particular, vibration-based energy harvesters have been intensively studied and developed because vibration energy sources exist almost everywhere in our daily life and industrial environment, and its conversion mechanism is relatively simple.

The early design of vibration energy harvesters was based on a linear resonator coupled with electromagnetic, electrostatic, or piezoelectric transducers [1,2]. Such linear-resonant energy harvesters produce high electric power only in a narrow resonant frequency band, which is not suitable for harvesting energy from real-world vibration sources with time-varying and/or multiple frequency components [3,4]. Accordingly, energy harvesting systems of various structures (e.g., frequency-tunable oscillators [5,6,7], an array of multimodal oscillators [8,9,10], and non-linear mono-stable [11,12,13,14] or multi-stable oscillators [15,16,17,18,19,20,21,22,23,24,25,26]) have been investigated to deal with the narrow bandwidth problem of the linear system.

A bi-stable oscillator that possesses a double-well potential energy function is known to be one of the most advantageous candidates to overcome the limitation of the linear system. The dynamic behavior of the bi-stable oscillator is generally more complicated than that of mono-stable oscillators owing to the existence of a saddle barrier between the potential energy wells, but its large-amplitude oscillation (called interwell motion) tends to cover a much broader frequency range. During the past decade, various types of bi-stable energy harvester have been proposed based mainly on magnetically (repulsive/attractive) and/or mechanically buckled structures, and their performance has been evaluated under ambient vibration sources of the swept sine, impulse, and Gaussian white noise types [1,2,18]. The performance of a bi-stable energy harvester depends on the amount of ambient vibration energy transmitted to the energy transducer, that is, transmissibility, which is related to the features of a forced interwell motion, such as the required intensity of excitation, the associated frequency bandwidth, and its oscillation frequency and amplitude. Various optimization strategies have been proposed to enhance the performance of vibration energy harvesters (e.g., artificial intelligence-based optimizations, structural optimizations, and nonlinear optimizations) [27,28]. Additionally, an external load resistance connected to an energy harvester for harvesting electrical energy is also an important design factor that should be optimized for maximizing performance. The resistance-matching technique has been commonly used for estimating the optimal load resistance [29], but it applies in principle to systems where both the source and load devices are linear. For non-linear systems (including a bi-stable system considered in this study), the resistance-matching technique may give an inaccurate estimation of the optimal load resistance with significant error, depending on the system and the excitation conditions.

In this study, a semi-analytic approach based on the harmonic balance method is proposed to optimize the external load resistance of a bi-stable electromagnetic energy harvester accurately, when compared with conventional resistance-matching techniques. To this end, the frequency responses of the primary harmonic and subharmonic interwell motions are numerically obtained first, and their solution forms are assumed in accordance with the results of spectral analyses. For each interwell motion, an optimization problem was formulated and solved, based on the harmonic balance solution of the assumed Fourier series, to calculate the optimal external load resistance of the energy harvester. In addition, a parametric study was conducted to investigate the effects of the system parameters and excitation conditions on the optimal load resistance of the electromagnetic energy harvester, which is not considered in conventional resistance-matching techniques; the results are discussed.

## 2. Bi-Stable Oscillator Model of an Electromagnetic Energy Harvester

**Figure 1:**
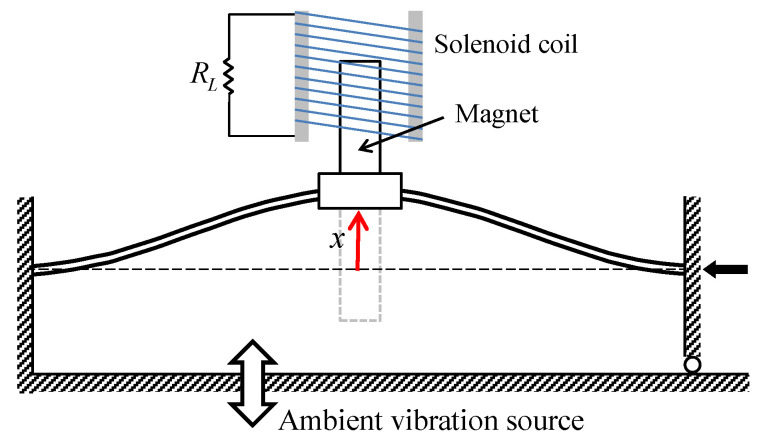
Schematic diagram of an electromagnetic bi-stable energy harvester composed of a clamped-clamped beam and an external solenoid coil. The right end of the beam is movable in the horizontal direction but can also be fastened at a certain position. The gravitational field is assumed to be in the perpendicular direction to the given plane.

Figure 1 shows the schematic diagram of an electromagnetic energy harvester considered in this study, which is composed of two parts: a clamped-clamped beam oscillator and an external solenoid coil structure. The inertial mass, along with two permanent magnets, is attached to the middle of the stainless-steel beam to adjust the natural frequency of the beam oscillator. The solenoid coil structure is fixed to the external space and aligned with the inertial mass, so that the permanent magnet can oscillate through the inner hole of the solenoid coil when the base of the energy harvesting system is excited by a certain ambient vibration. The coil is connected to an external load resistance for harvesting electrical energy. With this structure for electromagnetic induction, the energy harvesting system can convert ambient vibration energy to electrical energy that would be used (or stored in a capacitor) for powering micro-devices.

The performance of the energy harvesting system depends significantly on the transmissibility of the system, that is, the amount of ambient vibration transmitted through the beam oscillator to the permanent magnet traveling in the inner hole of the coil. Thus, the electromagnetic energy harvester is designed based on a statically bi-stable oscillator to better respond to various types of ambient vibration with wide-band frequency components. To this end, an axial load is imposed on the beam in the longitudinal direction by moving the right clamped end slightly to the left, which causes the buckling phenomenon of the beam. It is well known that at the moment when buckling occurs, new symmetric non-trivial equilibria ($x=\pm x_{0}$) start bifurcating from the trivial one (x=0), which is called the pitch-fork bifurcation of equilibrium. After the buckling, the beam structure becomes unstable at the trivial equilibrium, and is stable at the two non-trivial upper and lower equilibria depicted in Figure 1. 

The mathematical model for the aforementioned energy harvesting system is given by the following electro-magneto-mechanical oscillator model [30,31,32]:

(1a) $$\ddot{x}+2\zeta \omega_{0}\dot{x}-\gamma x_{0}^{2}x+\gamma x^{3}+\kappa_{0}I=-A_{b}\cos\Omega t,$$

(1b) $$L_{0}\dot{I}+\left(R_{e}+r_{L}\right)I=\beta \dot{x},$$

where x is the displacement of the inertial mass attached to the middle of the beam; $x_{0}$ indicates the distance of the non-trivial equilibria measured from the trivial one; γ is the coefficient given by ${\left(\omega_{0}/4x_{0}\right)}^{2}$; I is the electrical current flowing across the load resistance $R_{e}$; $\omega_{0}$ and ζ are the natural angular frequency and damping ratio, respectively; $\kappa_{0}$ and β are the electro-magneto-mechanical coupling constants; $r_{L}$ and $L_{0}$ are the internal resistance and inductance of the coil, respectively; and $A_{b}$ and Ω are the magnitude and frequency of the base acceleration, respectively. The baseline parameter values of the oscillator model are listed in Table 1. In addition, for the purpose of generality, the following dimensionless variables and parameters are introduced to Equations (1a) and (1b):

(2) $$y=\frac{x}{x_{0}},\;J=\frac{I}{I_{0}},\;\tau =\omega_{0}t,\;A=\frac{A_{b}}{x_{0}\omega_{0}^{2}},\;\omega_{r}=\frac{\omega }{\omega_{0}},\;\kappa =\frac{\kappa_{0}\beta L_{0}}{\alpha^{2}{r_{L}}^{2}},\;\alpha =\frac{\omega_{0}L_{0}}{r_{L}},\;R=\frac{R_{e}}{r_{L}},$$

and the resulting dimensionless forms of governing equations can be obtained in the form:

(3a) $$\ddot{y}+2\zeta \dot{y}-\frac{1}{2}y+\frac{1}{2}y^{3}+\kappa J=-A\cos\omega \tau ,$$

(3b) $$\alpha \dot{J}+\left(R+1\right)J=\dot{y},$$

where ω are and the dimensionless forcing frequency (or the frequency ratio); α is the dimensionless resonant inductive reactance (or the ratio of the resonant inductive reactance of the coil, $\omega_{0}L_{0}$, to the internal resistance of the coil, $r_{L}$); and R is the dimensionless external load resistance (or the ratio of the external load resistance to the internal resistance of the coil).

## 3. Numerical Frequency Response Analysis of an Electro-Magneto-Mechanical Oscillator Model 

### 3.1. Steady-State Response and Its Bifurcation

In this work, a series of numerical simulations of the electro-magneto-mechanical oscillator model expressed in Equations (3a) and (3b) are performed to investigate the dynamic behaviors of the system and the associated energy harvesting performance. By introducing the state vector $\left[y_{1},y_{2},y_{3},y_{4}\right]=\left[\dot{y},y,J,\theta \right]$, where θ=ωτ, into Equations (3a) and (3b), the dimensionless governing differential equations can be rewritten into an autonomous system of first-order differential equations in the following vector form:

(4) $$\dot{y}=f(y,t)=\left[\begin{array}{c} -2\zeta y_{1}+\frac{1}{2}y_{2}-\frac{1}{2}y_{2}^{3}-\kappa y_{3}-A\cos y_{4}\\ y_{1}\\ -\frac{R+1}{\alpha }y_{3}+\frac{1}{\alpha }y_{1}\\ \omega \end{array}\right].$$

Frequency response analyses are carried out by the direct numerical integration of Equation (4) based on the Runge–Kutta methods. For all the numerical simulations, the baseline parameter values given in Table 1 are used, unless otherwise stated. The steady-state forced responses of the electromagnetic energy harvester are evaluated first, while the frequency of the base excitation varies by means of frequency marching or frequency sweeping, and then their Floquet multipliers are obtained by solving the eigenvalue problem of the following monodromy matrix:

(5) $$M=\prod_{i=N}^{1}\exp\left[\int_{\tau_{i-1}}^{\tau_{i}}J(\tau )d\tau \right],$$

where J is the Jacobian matrix, and *N* is the number of equally spaced time intervals included within one period. The bifurcation type of the stationary oscillation can be determined from the investigation of its Floquet multipliers. When the stability of the steady-state solution changes, the accompanying bifurcations can be classified into three different types: saddle-node bifurcation (if the largest Floquet multiplier μ is positive 1, i.e., μ=+1), period-doubling bifurcation (μ=−1), and Neimark–Sacker bifurcation (|μ|=1 and Im[μ]≠0). 

### 3.2. Non-Linear Oscillations Owing to Double-Well Potential: Intrawell and Interwell Motions

**Figure 2:**
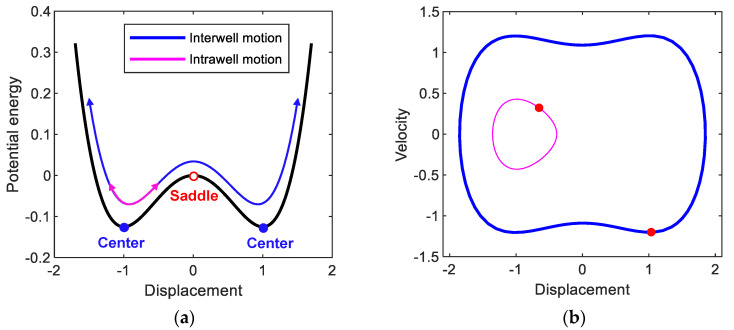
(**a**) Double-well potential energy function of the electromagnetic bi-stable energy harvester. The open and solid circles indicate the saddle and center points, respectively. (**b**) Phase plane representation of the intrawell and interwell oscillations (of period T=2π/ω), which are denoted by thick (blue) and thin (magenta) lines, respectively, obtained when *A* = 0.02 and *ω* = 0.82. In (**b**), the red points indicate the stroboscopic projections of the oscillations, which are synchronized with the period *T* of the base excitation.

The electromagnetic bi-stable energy harvester possesses a double-well potential energy function, as shown in Figure 2a. There are one trivial saddle point and two non-trivial center points in the potential function. The two potential wells are in symmetric configuration with respect to the potential saddle barrier formed at the trivial point of y=0. This potential saddle barrier leads to two different types of forced response: intrawell and interwell motions, depending on the intensity of the base excitation. When the base excitation is weak, the system only oscillates with small amplitude inside one of the two potential wells (as demonstrated by the thin line in Figure 2b), which is called intrawell motion, owing to the existence of the potential barrier. When the base excitation becomes relatively strong, the system gains enough kinetic energy to cross the potential barrier (the thick line in Figure 2b). Accordingly, it begins oscillating with a much larger amplitude, producing higher electrical power, and it is called interwell motion. The interwell motion tends to occur over a much broader frequency band than the resonant frequency band of linear oscillators. Using this interwell motion, the electromagnetic bi-stable energy harvester can harvest energy from ambient vibration energy sources, including wide-band frequency components. The aforementioned high-energy interwell motion of the electromagnetic energy harvester appears in various types of oscillations: primary harmonic motion, its subharmonic and superharmonic motions, and even non-periodic chaotic motion. Among these interwell motions, the primary harmonic motion of period T=2π/ω has been used the most for broadband energy harvesting applications. However, it was recently reported that subharmonic motions are also useful and suitable [29].

**Figure 3:**
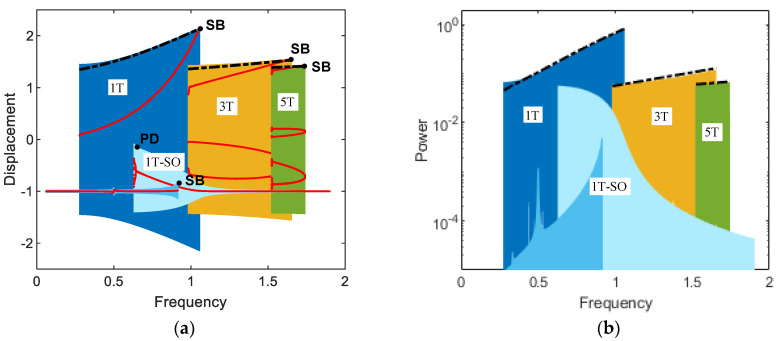
Frequency responses for (**a**) displacement and (**b**) output power of the electromagnetic bi-stable energy harvester obtained when *A* = 0.02: (1*T*) primary harmonic period-*T* interwell motion, (1*T*-SO) period-*T* intrawell motion, (3*T*) subharmonic period-3*T* interwell motion, and (5*T*) period-5*T* interwell motion. The saddle-node bifurcations and period-doubling bifurcation are designated by points SB and PD, respectively. In (**a**), the red dots indicate the stroboscopic projections of the oscillations, which are synchronized with the period *T* of the base excitation. The dashed-dotted lines are the harmonic balance solutions for the primary harmonic and subharmonic interwell motions, which will be discussed in Section 4.

**Figure 4:**
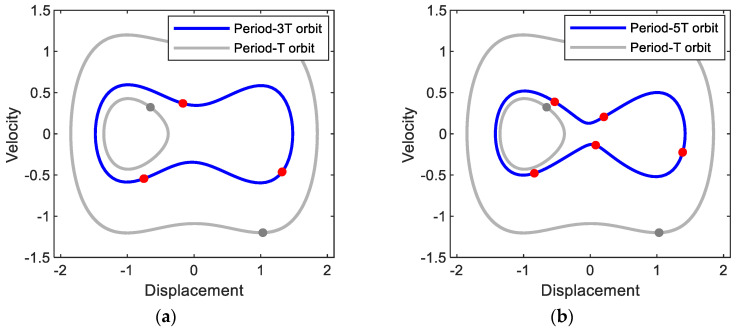
Phase plane representations of (**a**) period-3*T* and (**b**) period-5*T* motions obtained with the base acceleration of *A* = 0.02 and forcing frequencies of (**a**) *ω* = 1.37 and (**b**) *ω* = 1.65, respectively. The period-*T* interwell and intrawell motions of Figure 2b are also presented for comparison purpose. The solid circle dots indicate the stroboscopic projections of the oscillations, which are synchronized with the period *T* of the base excitation.

Figure 3 shows the frequency responses for (a) displacement and (b) output power of the electromagnetic energy harvester obtained when the base acceleration is 0.02. In this figure, the subharmonic interwell motions of periods 3*T* and 5*T* and the primary harmonic interwell motion of period *T* are presented along with the intrawell motion for comparison purposes. The subharmonic interwell motions are much larger in amplitude than the intrawell motion (as shown in Figure 3a), although they are smaller than the primary harmonic interwell motion, and accordingly can produce relatively higher output powers (as shown in Figure 3b). This is more clearly demonstrated in Figure 4 by directly comparing the period-1*T*, 3*T*, and 5*T* interwell motions on the state plane. The period-3*T* interwell motion has a notably wide frequency band, similar to that of the period-*T* interwell motion. It is interesting that the frequency bands of the period-1*T*, 3*T*, and 5*T* interwell motions are continuously connected to each other and tend to form a whole wide frequency band. The above observations indicate that the subharmonic interwell motions in addition to the primary harmonic interwell motion can play an important role in enhancing the operating frequency band of electromagnetic energy harvesters.

## 4. Semi-Analytic Approach to Optimizing the External Resistance

The external load resistance of an electromagnetic energy harvester, together with the transmissibility, is an important design factor, which must be optimized to maximize its output electrical power. An impedance-matching technique has been commonly used to solve this optimization problem, with the assumption that the inductance of the coil is negligible. In this case, the external load resistance is actually matched with the internal resistance of the solenoid coil; thus, it will be called ‘resistance matching’ in this study. However, it should be noted that the impedance- (or resistance-) matching technique only applies when both the source and load devices are linear, although they are frequently used for non-linear systems at the cost of accuracy. In this work, a semi-analytical approach based on the method of harmonic balance is implemented to evaluate the optimal value of the external load resistance accurately, with which the interwell motion of the system can most efficiently produce electrical power.

**Figure 5:**
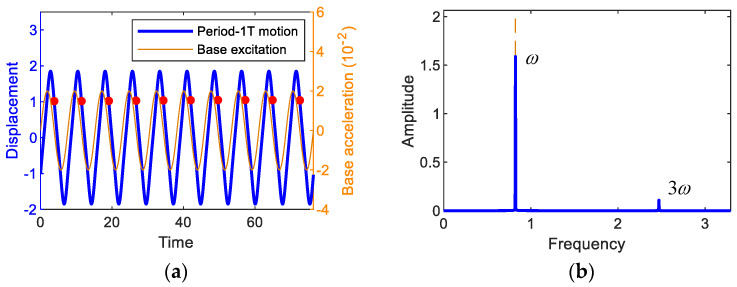

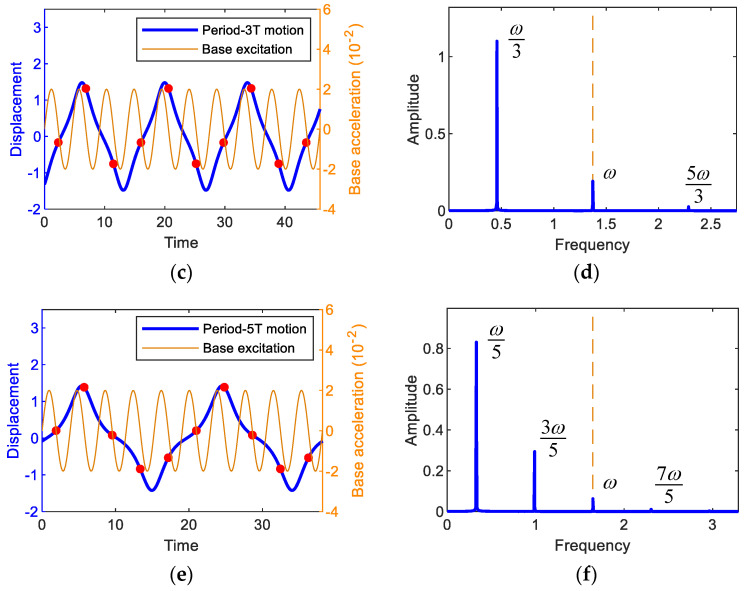
(**first column**) Time responses and (**second column**) the associated amplitude spectra, obtained by the fast Fourier transform. In (**a**,**b**) the period-1*T* motion when *A* = 0.02 and *ω* = 0.82, in (**c**,**d**) the period-3*T* motion when *A* = 0.02 and *ω* = 1.37, and in (**e**,**f**) the period-5*T* motion when *A* = 0.02 and *ω* = 1.65. The base excitation used for this simulation is also presented for comparison. In (**a**,**c**,**e**), the red dots indicate the stroboscopic projections of the oscillations, which are synchronized with the period *T* of the base excitation.

First, Fourier analyses are performed to find the appropriate solution forms of the steady-state primary harmonic and subharmonic interwell motions, which depend on the type and intensity of non-linearity [33], and the results of the time responses and the corresponding amplitude spectra are presented in Figure 5. The forcing period of the base excitation is given by T=2π/ω, and accordingly the oscillation frequencies of the subharmonic period-*3T* and 5*T* motions tend to be lower than the forcing frequency *ω*, as shown in Figure 5c,e. Such subharmonic behavior is known to be a typical feature of a bi-stable system. Therefore, the fundamental oscillation frequencies of the interwell motions can be defined by $\omega_{k}=\omega /k$, where *k* = 1, 3, and 5 for the period-1*T*, *3T*, and 5*T* motions, respectively. From Figure 5b,d,f, it can be observed that the interwell motions only have two or three important frequency components: $\omega_{1}$- and $3\omega_{1}$-components for the period-*T* motion, $\omega_{3}$- and $3\omega_{3}$-components for the period-*3T* motion, and $\omega_{5}$-, $3\omega_{5}$- and $5\omega_{5}$-components for the period-5*T* motion, as shown in Figure 5b,d,e, respectively. Thus, the steady-state periodic solutions can be assumed in the following forms of the *N*th-order Fourier series:

(6a) $$(\mathrm{Period}-T\;\mathrm{motion})\;y=\sum_{n=1,3}a_{n}\cos n\omega \tau +b_{n}\sin n\omega \tau ,\;J=\sum_{n=1,3}c_{n}\cos n\omega \tau +d_{n}\sin n\omega \tau$$

(6b) $$(\mathrm{Period}-3T\;\mathrm{motion})\;y=\sum_{n=1,3}a_{n}\cos\frac{n\omega }{3}\tau +b_{n}\sin\frac{n\omega }{3}\tau ,\;J=\sum_{n=1,3}c_{n}\cos\frac{n\omega }{3}+d_{n}\sin\frac{n\omega }{3}$$

(6c) $$(\mathrm{Period}-5T\;\mathrm{motion})\;y=\sum_{n=1,3,5}a_{n}\cos\frac{n\omega }{5}\tau +b_{n}\sin\frac{n\omega }{5}\tau ,\;J=\sum_{n=1,3,5}c_{n}\cos\frac{n\omega }{5}+d_{n}\sin\frac{n\omega }{5}$$

where the highest order of harmonics is given as *N* = 3 for the period-1*T* and 3*T* motions and *N* = 5 for the period-5*T* motion, and all the constant terms are set to zero as the main interest is on the interwell motion, of which the center of oscillation is the trivial point (*y* = 0). 

**Figure 6:**
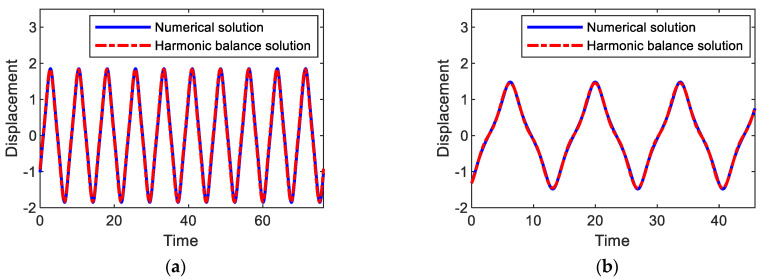

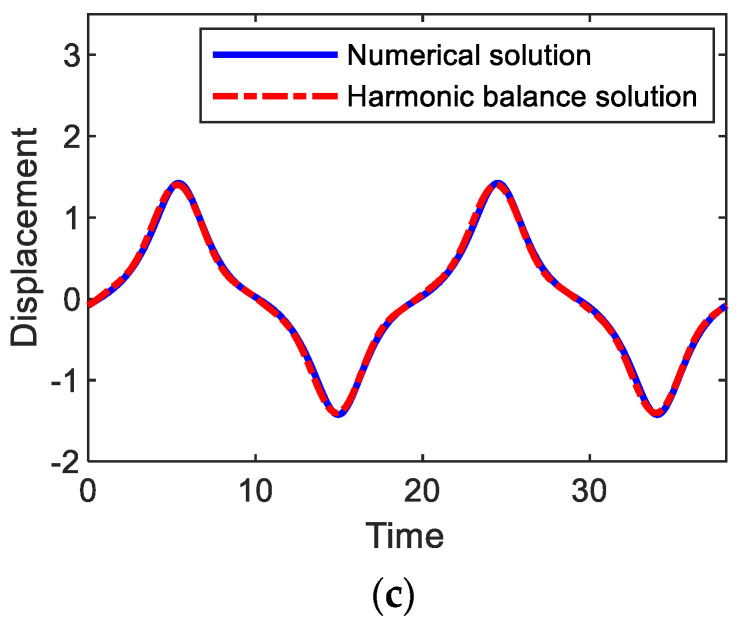
Comparisons between numerical and analytical solutions for (**a**) period-1*T*, (**b**) 3*T*, and (**c**) 5*T* interwell motions, which are obtained when the amplitude and frequency of the base acceleration are given as *A* = 0.02 and *ω* = (**a**) 0.82, (**b**) 1.37, and (**c**) 1.65.

Substituting Equations (6a)–(6c) into Equations (3a) and (3b), followed by equating the coefficients of like harmonic components, leads to a system of non-linear algebraic equations for unknown variables $a_{n}$, $b_{n}$, $c_{n}$, and $d_{n}$, which are numerically solved by the Newton–Raphson method. As shown in Figure 3, the harmonic balance solutions (dashed-dotted lines) for primary harmonic and subharmonic interwell motions are well matched with the numerical solutions. Additionally, time-domain comparisons are made in Figure 6, where numerical and analytical results are also in a good agreement with each other.

Using the obtained harmonic balance solutions, the average output power $P_{av}$ of the electromagnetic energy harvester can be evaluated as $P_{av}^{2}=\sum \left(c_{n}^{2}+d_{n}^{2}\right)/2$, and the optimal value of the external load resistance is estimated by solving the optimization problem of $\partial P_{av}/\partial R=0$. The optimal external load resistance reduces to 1 for the same problem when the resistance matching technique is used.

**Figure 7:**
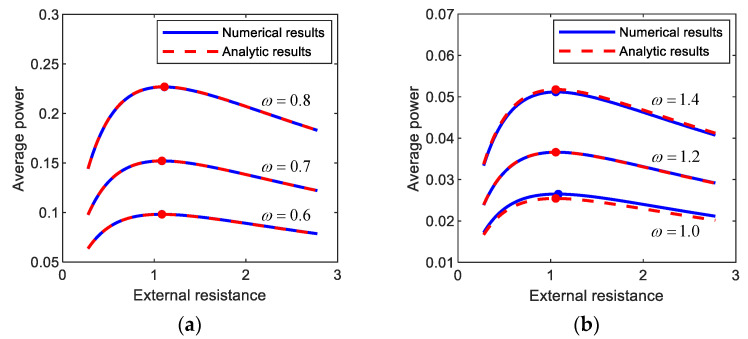

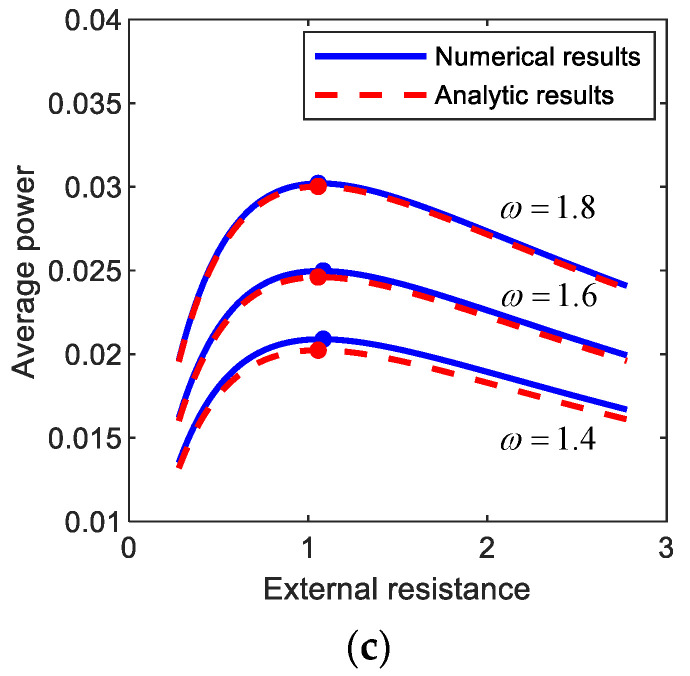
Plots of the external load resistance versus the average output power harvested from (**a**) period-1*T*, (**b**) period-3*T*, and (**c**) period-5*T* motions of the electromagnetic energy harvester. The results are obtained by numerical integration (blue solid lines) and the semi-analytic approach (red dashed lines) and compared to each other. For each motion, the comparisons in between are made with three different forcing conditions: (**a**) (*A*, *ω*) = (0.012, 0.6), (0.02, 0.7), (0.03, 0.8), (**b**) (*A*, *ω*) = (0.012, 1.0), (0.02, 1.2), (0.03, 1.4), (**c**) (*A*, *ω*) = (0.012, 1.4), (0.02, 1.6), (0.03, 1.8). The solid circle points indicate the optimal conditions for the external load resistance with which the output power is maximized.

Figure 7 shows the average output powers, harvested from (a) period-1*T*, (b) period-3*T*, and (c) period-5*T* motions, with respect to the external load resistance. The numerical and semi-analytical results are compared to each other in Figure 7. As shown in this figure, the analytic and numerical results for the average output powers are well matched with each other. Furthermore, the optimal values of the external load resistance were evaluated, and it was observed that the analytic results are in an excellent agreement with numerical results with a maximum relative error of less than (a) 0.3%, (b) 2.0%, and (c) 1.5%. This observation supports the validity of the semi-analytic approach to optimize the external load resistance implemented in this study.

In general, the subharmonic period-3*T* and 5*T* interwell motions tend to produce relatively lower electric power than the primary harmonic period-1*T* interwell motion (but relatively higher than intrawell motions). However, the frequency bands of the period-1*T*, 3*T*, and 5*T* interwell motions are possibly different from each other (as shown in Figure 3), and each of the interwell motions could be used to harvest energy in the associated frequency band, depending on excitation conditions.

## 5. Effects of Inductive Reactance and Excitation Conditions on Optimal External Resistance

Using the semi-analytic method mentioned in the previous section, a parametric study is conducted to investigate the effects of the dimensionless resonant inductive reactance α (defined by the ratio of $\omega_{0}L_{0}$ to $r_{L}$ in Equation (3b)) and the excitation conditions (the frequency and intensity of the base excitation) on the optimal load resistance of the electromagnetic energy harvester, which is not considered in the resistance-matching technique. 

**Figure 8:**
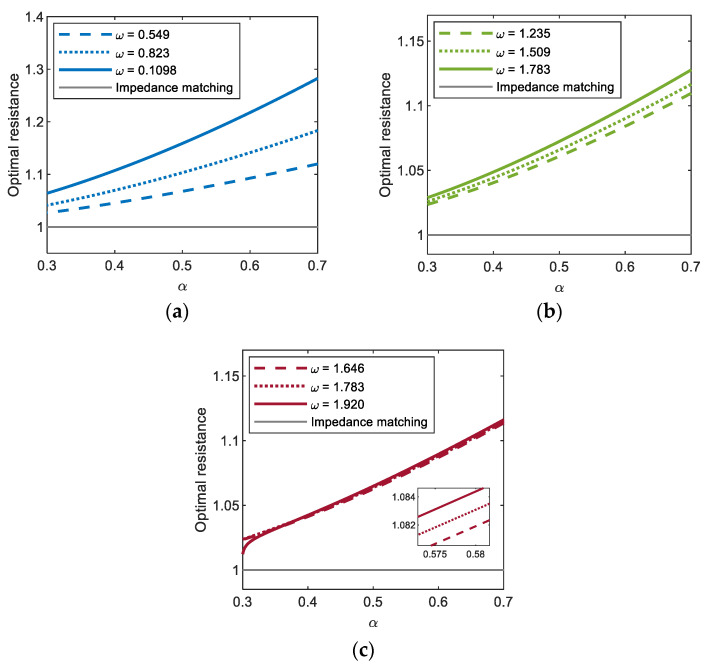
Plots of the resonant inductive reactance versus the optimal load resistance for (**a**) period-1*T*, (**b**) period-3*T*, and (**c**) period-5*T* motions of the electromagnetic energy harvester. For each motion (colored thick lines), the results are calculated by using the semi-analytic method, with three different values of excitation frequency and a constant base acceleration of 0.02, and compared with that obtained by the resistance-matching technique (grey thin line).

Figure 8 shows the optimal values of the external load resistance for (a) period-1*T*, (b) period-3*T*, and (c) period-5*T* motions of the electromagnetic energy harvester, obtained by using the semi-analytic method, with respect to the resonant inductive reactance. In this figure, the results clearly indicate that the optimal load resistance of the electromagnetic energy harvester can be significantly affected by the resonant inductive reactance and excitation frequency. For all the period-1*T*, 3*T*, and 5*T* motions, the optimal load resistance increases as the dimensionless resonant inductive reactance increases––in other words, as the inductance ($L_{0}$) of the solenoid coil becomes relatively larger––and accordingly, it deviates from the optimal value (i.e., 1) estimated by the resistance-matching technique, in which the inductance of the coil is assumed to be zero. Such a deviation tends to be larger when the frequency (ω) of the base excitation is higher, which is more remarkable for the interwell motion of the shorter oscillation period (in the order of periods (a) 1*T*, (b) 3*T*, and (c) 5*T* in Figure 8) or higher oscillation frequency (in the order of (a) $\omega_{1}$, (b) $\omega_{3}$, and (c) $\omega_{5}$). From these observations, it is deduced that the optimal load resistance depends on the inductive reactance of the coil, which is defined by $\omega_{k}L_{0}$, in addition to the internal resistance ($r_{L}$) of the coil. 

**Figure 9:**
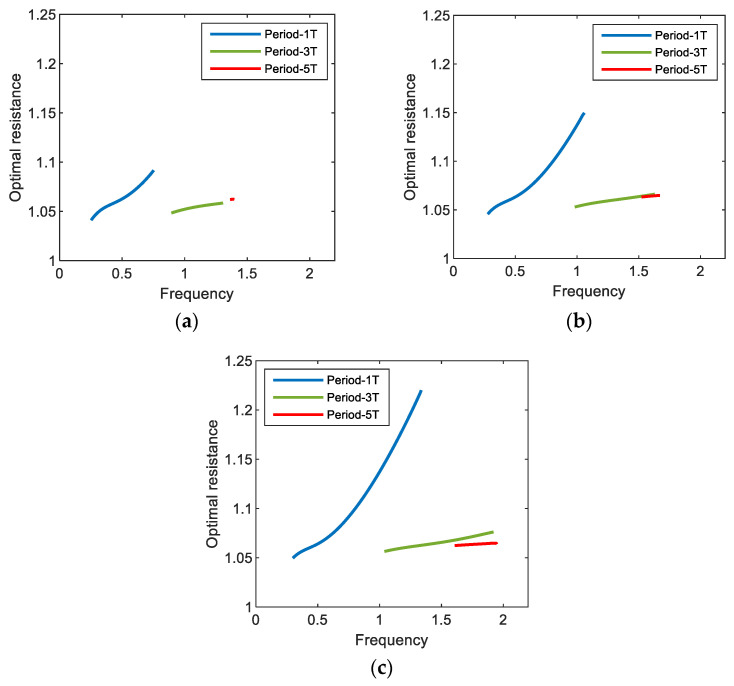
Plots of the excitation frequency versus the optimal load resistance for the interwell motions (with periods 1*T*, 3*T*, and 5*T*) of the electromagnetic energy harvester. In this simulation, the acceleration of base excitation is set to (**a**) 0.012, (**b**) 0.02, and (**c**) 0.03.

**Figure 10:**
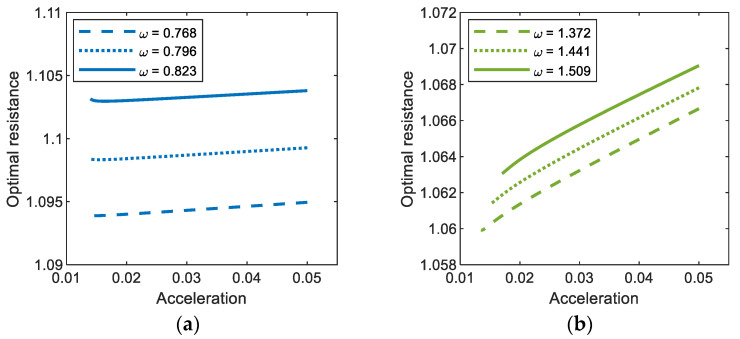

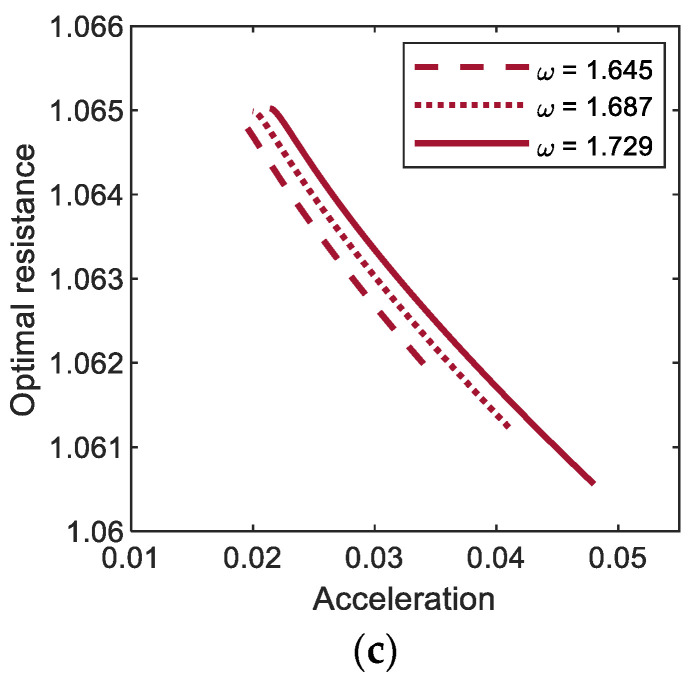
Base acceleration versus optimal load resistance for (**a**) period-1*T*, (**b**) period-3*T*, and (**c**) period-5*T* motions of the bi-stable electromagnetic energy harvester.

Figure 9 shows the optimal values of the external load resistance for the period-1*T*, 3*T*, and 5*T* motions of the electromagnetic energy harvester, obtained with three different values of base acceleration: (a) *A* = 0.012, (b) *A* = 0.02, and (c) *A* = 0.03. The calculations are made only in the range of the excitation frequency in which the interwell (period-1*T*, 3*T*, and 5*T*) motions exist. As shown in this figure, the period-1*T* and period-3*T* motions occur in a broad range of excitation frequencies, whereas the period-5*T* motion occurs in a relatively narrow range, which means that the former are more important design factors in broadband energy harvesting applications. The frequency range for each interwell motion tends to be broader as the intensity of the base excitation increases, slightly shifting to a higher frequency. For the period-1*T* motion, the optimal load resistance obviously increases with excitation frequency, whereas for other interwell motions, only small variations in the optimal load resistance are observed in the overall range of excitation frequency (particularly for period-5*T* motion, the resistance is almost constant). This is because the lower the oscillation frequencies of the subharmonic period-3*T* and 5*T* motions ($\omega_{3}=\omega /3$ and $\omega_{5}=\omega /5$, respectively) are, the weaker the effects of the inductive reactance of the solenoid coil on the optimal resistance. Furthermore, the optimal load resistance is not sensitive to changes in the intensity of the base excitation. As shown in Figure 10, the optimal load resistance for the period-1*T* and 3*T* motions tends to increase slightly with the base acceleration and, contrarily, the one for the period-5*T* motion decreases slightly. However, these changes in the optimal resistance are not significant (less than 0.01%).

## 6. Broadband Energy Harvesting Applications

**Figure 11:**
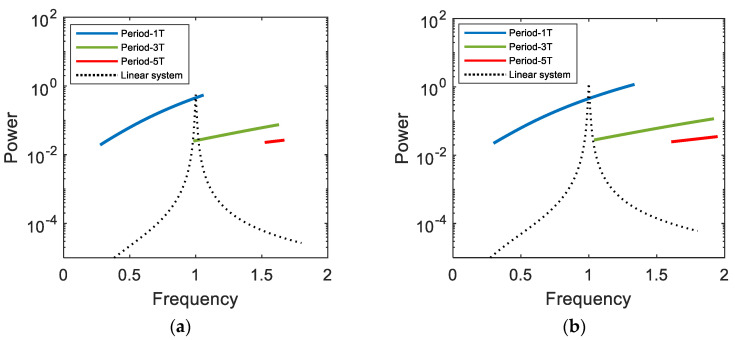
Frequency marching responses for the output powers of the electromagnetic bi-stable energy harvester and its linear counterpart, which are obtained when (**a**) *A* = 0.02 and (**b**) 0.03.

The output power of the bi-stable electromagnetic energy harvester is evaluated, while the frequency of the harmonic base excitation varies by means of frequency marching, and compared with that of its linear counterpart in Figure 11 to demonstrate its broadband energy harvesting performance. As shown in this figure, for the linear system, a sharp resonant peak of the output power response appears in a very narrow frequency band. In this case, the natural frequency of the system must be designed to be synchronized with the forcing frequency of the harmonic excitation, otherwise it is likely to fail to produce high output power. On the contrary, the bi-stable system possesses the multiple branches of the interwell motions (including the period-1*T*, 3*T*, and 5*T* motions) in a broad frequency band and thereby can keep normally operating with high output power, regardless of the synchronization of the frequencies. Therefore, the bi-stable energy harvester is definitely more advantageous in real applications, in which the ambient harmonic vibration possibly varies, than the linear system. Actually, there are various types of ambient vibration source such as harmonic, periodic, impulsive, and random excitations. The external load resistance of the bi-stable electromagnetic energy harvester should be optimized in an appropriate manner that depends on the type of the ambient vibration source.

Basically, the semi-analytical optimization approach proposed in this study is suitable for harmonic vibration sources, since it is based on the harmonic balance solution. For all the above simulations, the optimal load resistance was evaluated and investigated, when the frequency of the harmonic base excitation was constant, to demonstrate the dynamic characteristics of the optimal resistance. In this section, the proposed optimization approach is further extended for piecewise harmonic excitations with constant amplitude but stepwise changes in frequency. 

The general form of the average output power is given in the form:

(7) $$P_{av}=J_{RMS}^{2}R=\frac{1}{\Delta T}\int_{T_{0}}^{T_{N}}J^{2}Rd\tau ,$$

where $J_{RMS}$ is the root mean square (RMS) of the output current flowing across the external load resistance over the whole-time interval $\Delta T(=T_{n}-T_{0})$. For most base excitations, the numerical integration method can be commonly used to evaluate the average power of Equation (7). Particularly for the piecewise harmonic excitation, the frequency of which is defined on a sequence of sub-time intervals $\Delta T_{k}=T_{k}-T_{k-1}\;(k=1,2,\cdots ,N)$, the resulting average output power is rewritten as the weighted sum of *N* RMS values:

(8a) $$P_{av}=\frac{\Delta T_{1}}{\Delta T}\left(\frac{1}{\Delta T_{1}}\int_{T_{0}}^{T_{1}}J^{2}Rd\tau \right)+\frac{\Delta T_{2}}{\Delta T}\left(\frac{1}{\Delta T_{2}}\int_{T_{1}}^{T_{2}}J^{2}Rd\tau \right)+\cdots +\frac{\Delta T_{N}}{\Delta T}\left(\frac{1}{\Delta T_{N}}\int_{T_{N-1}}^{T_{N}}J^{2}Rd\tau \right),$$

or

(8b) $$P_{av}=\sum_{k=1}^{n}r_{k}J_{k}^{2}R\;\mathrm{with}\;r_{k}=\frac{\Delta T_{k}}{\Delta T}\;\mathrm{and}\;J_{k}=\sqrt{\frac{1}{\Delta T_{k}}\int_{T_{k-1}}^{T_{k}}J^{2}d\tau },$$

where $J_{k}$ is the RMS output current on the subinterval $\Delta T_{k}$, and $r_{k}$ is the weighting factor. Assuming that the base excitation is harmonic on each subinterval $\Delta T_{k}$, the RMS output current ($J_{k}$) and the associated average power ($J_{k}^{2}R$) can be evaluated by using the harmonic balance solutions of Equations (6a)–(6c). The optimization problem for maximizing the total output power given by Equation (8) is readily formulated and solved to estimate a single optimal value of the external load resistance.

**Figure 12:**
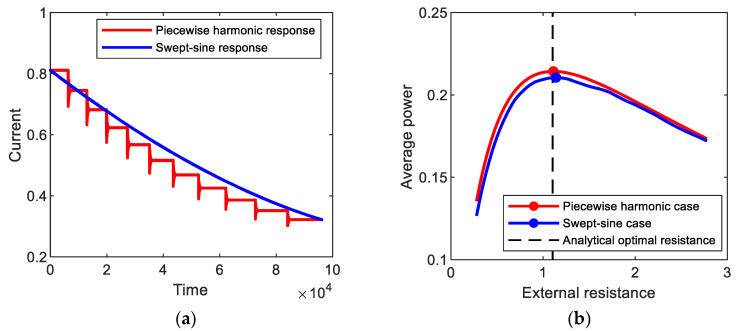
(**a**) Amplitudes of the output current responses to (red line) piecewise harmonic and (blue line) swept-sine excitations, which are obtained for the period-1*T* interwell motions of the bi-stable electromagnetic energy harvester, and (**b**) average output powers evaluated with the variation of the external load resistance.

Figure 12a shows the amplitude of the output current response to (red line) piecewise harmonic excitation, which is obtained numerically for the period-1*T* interwell motion of the bi-stable electromagnetic energy harvester. In this simulation, the forcing frequency of the piecewise base excitation decreased from 1.0 by 0.05 to 0.5 for every 1000 cycles, but the amplitude of the base acceleration is set to be 0.02 g. The resulting output current response tends to intermittently vary with stepwise change in excitation frequency. Additionally, the average output power is evaluated with the variation of the external load resistance, as illustrated in Figure 12b. The optimal load resistance, 1.111, is estimated and compared with the analytical one, 1.104, evaluated by using Equations (6) and (8). The relative error of the analytical optimal resistance is very small (less than 0.7%), which supports the assertion that the proposed optimization approach is also valid for the piecewise harmonic excitation. In Figure 12a,b, the results for (blue line) swept-sine excitation are presented for comparison purposes. The swept-sine excitation continuously decreased with the slow sweep rate of 5 × 10^−6^ in the same frequency range. The output current response to the swept-sine excitation gradually decreases in the similar tendency to the piecewise harmonic case but with the difference in amplitude, which results in the difference in average output power. However, the optimal resistance, 1.139, for the swept-sine excitation (particularly with a slow sweep rate) is very similar to that for the piecewise harmonic case, with a small difference (less than 2.6%). This means that the semi-analytical optimal resistance is possibly used as an approximate for slowly swept excitation.

**Figure 13:**
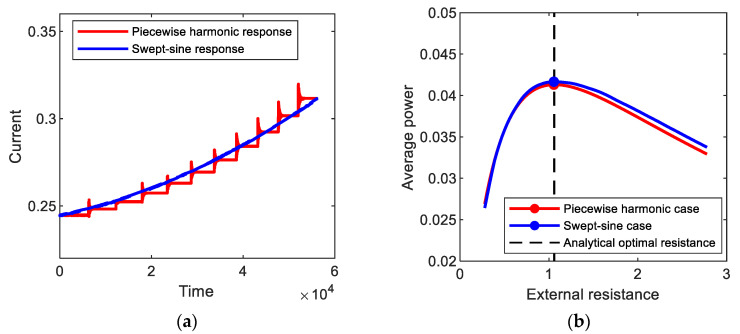
(**a**) Amplitudes of the output current responses to (red line) piecewise harmonic and (blue line) swept-sine excitations, which are obtained for the period-3*T* interwell motions of the bi-stable electromagnetic energy harvester, and (**b**) average output powers evaluated with the variation of the external load resistance.

As another example, Figure 13 shows the output current responses to piecewise harmonic and swept-sine excitations, which is obtained numerically for the period-3*T* interwell motion. The forcing frequency of the piecewise base excitation increased from 1.0 by 0.05 to 1.5 for every 1000 cycles. The frequency of the swept-sine excitation increased with the sweep rate of 8.9 × 10^−6^. The numerical optimal resistances for the piecewise harmonic and swept-sine excitations are nearly close to each other, approximately 1.056. The semi-analytical optimal resistance, 1.059, is in good agreement with the numerical results for both the piecewise harmonic and swept-sine excitations.

## 7. Conclusions

In this study, a semi-analytic approach to optimizing the external load resistance of an electromagnetic energy harvester was proposed for primary harmonic (period-1*T*) and subharmonic (period-3*T* and 5*T*) interwell motions. The harmonic balance solutions of three interwell motions were obtained first and used to formulate an optimization problem for the external load resistance. The optimal load resistance was obtained, by solving the formulated problem, and investigated. A parametric study was performed to investigate the effects of the system parameters and excitation conditions on the optimal load resistance of the electromagnetic energy harvester. The results showed that for all the period-1*T*, 3*T*, and 5*T* motions, the optimal load resistance tended to increase as the inductance of a solenoid coil was larger and as the frequency of the base excitation was higher, but it was not sensitive to the intensity of the base excitation. Additionally, the optimal load resistance significantly depended on the oscillation frequency (or period) of the interwell motion—the higher the oscillation frequency (or the shorter the period) is, the larger the optimal resistance; thus, for the period-1*T*, 3*T*, and 5*T* motions in order. All of the above observations consistently indicate that the effect of the inductive reactance of the solenoid coil on the optimal load resistance becomes significant, particularly when the frequency of ambient vibration is relatively high. In this case, the non-linear dynamic behaviors of the interwell motions and the associated inductive reactance of the solenoid coil should be considered as important design factors in the optimization process of the electromagnetic energy harvester. Therefore, when compared with conventional resistance-matching techniques, the semi-analytic approach presented in this study could provide a more accurate estimation of the optimal load resistance.

## Figures and Tables

**Table 1:** Parameter values of the electro-magneto-mechanical oscillator [30,32].

Parameter	Value
$x_{0}$	0.29 mm
R	18 Ohm
$\omega_{0}$	365 Hz
ζ	0.004
$\kappa_{0}$	16.7
β	0.5
$r_{L}$	18 Ohm
$L_{0}$	5 mH

## Data Availability

Data sharing not applicable.
